# New Era of Diacylglycerol Kinase, Phosphatidic Acid and Phosphatidic Acid-Binding Protein

**DOI:** 10.3390/ijms21186794

**Published:** 2020-09-16

**Authors:** Fumio Sakane, Fumi Hoshino, Chiaki Murakami

**Affiliations:** Department of Chemistry, Graduate School of Science, Chiba University, Chiba 263-8522, Japan; f.hoshino@chiba-u.jp (F.H.); 12s3039@chiba-u.jp (C.M.)

**Keywords:** diacylglycerol kinase, phosphatidic acid, phosphatidic acid-binding protein, fatty acid, docosahexaenoic acid, phosphatidylinositol turnover, lipid sensor

## Abstract

Diacylglycerol kinase (DGK) phosphorylates diacylglycerol (DG) to generate phosphatidic acid (PA). Mammalian DGK consists of ten isozymes (α–κ) and governs a wide range of physiological and pathological events, including immune responses, neuronal networking, bipolar disorder, obsessive-compulsive disorder, fragile X syndrome, cancer, and type 2 diabetes. DG and PA comprise diverse molecular species that have different acyl chains at the *sn*-1 and *sn*-2 positions. Because the DGK activity is essential for phosphatidylinositol turnover, which exclusively produces 1-stearoyl-2-arachidonoyl-DG, it has been generally thought that all DGK isozymes utilize the DG species derived from the turnover. However, it was recently revealed that DGK isozymes, except for DGKε, phosphorylate diverse DG species, which are not derived from phosphatidylinositol turnover. In addition, various PA-binding proteins (PABPs), which have different selectivities for PA species, were recently found. These results suggest that DGK–PA–PABP axes can potentially construct a large and complex signaling network and play physiologically and pathologically important roles in addition to DGK-dependent attenuation of DG–DG-binding protein axes. For example, 1-stearoyl-2-docosahexaenoyl-PA produced by DGKδ interacts with and activates Praja-1, the E3 ubiquitin ligase acting on the serotonin transporter, which is a target of drugs for obsessive-compulsive and major depressive disorders, in the brain. This article reviews recent research progress on PA species produced by DGK isozymes, the selective binding of PABPs to PA species and a phosphatidylinositol turnover-independent DG supply pathway.

## 1. Introduction

**Figure 1:**
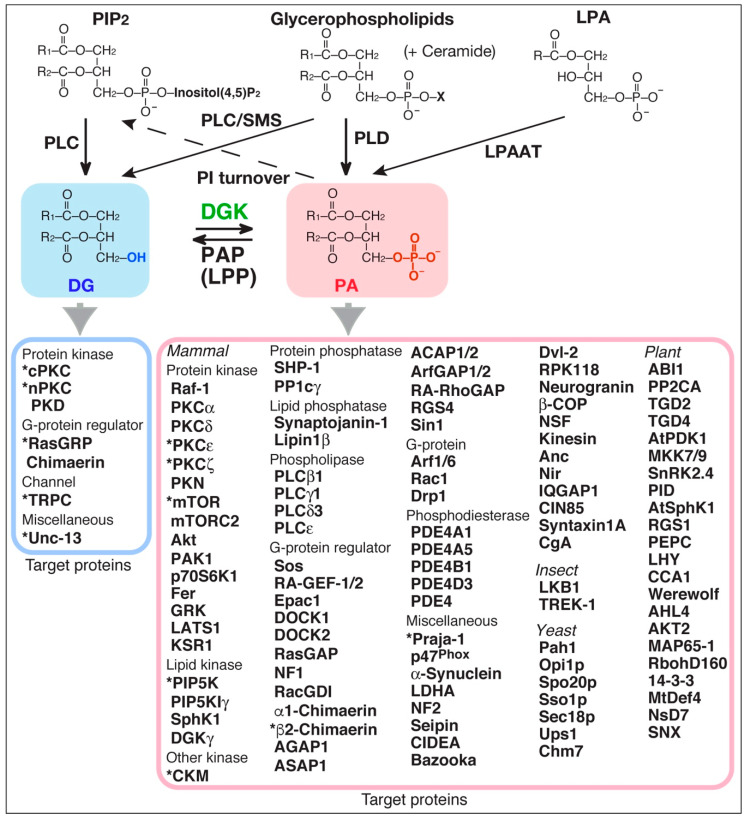
Target proteins of substrate (DG) and reaction product (PA) of DGK. Asterisks indicate target proteins that have been strongly suggested to be regulated by DGK isoforms through consumption of DG or production of PA. PAP, PA phosphatase; LPP, lipid phosphate phosphatase; PC, phosphatidylcholine; PI, phosphatidylinositol; PIP_2_, PI 4,5-bisphosphate; PLC, phospholipase C; PLD, phospholipase D; cPKC, conventional protein kinase C (PKCα, β and γ); nPKC, novel PKC (PKC δ, ε, η and θ); PKD, protein kinase D; Unc-13, uncoordinated-13; RasGRP, Ras guanyl nucleotide-releasing protein; TRPC, transient receptor potential channel; chimaerin (RacGAP); Raf-1/C-Raf, rapidly accelerated fibrosarcoma (serine/threonine protein kinase); PKCζ (atypical PKC (aPKC)); PKN (aPKC); mTOR, mammalian target of rapamycin (serine/threonine protein kinase); mTORC2, mTOR complex 2; Akt/protein kinase B; PAK1, p21-activated kinase 1; p70S6K1/S6K1, ribosomal protein S6 kinase β-1; Fer (tyrosine protein kinase); GRK, G protein-coupled receptor kinase; LATS1, large tumor suppressor kinase 1; KSR1, kinase suppressor of Ras 1 (serine/threonine protein kinase/scaffold protein); PIP5K, PI-4-phosphate 5-kinase; SphK1, sphingosine kinase 1; CKM, creatine kinase-muscle type; SHP-1, Src homology 2 domain-containing protein-tyrosine phosphatase 1; PP1c, protein phosphatase-1 catalytic subunit; synaptopjanin-1 (PI(4,5)P_2_-5-phosphatase); lipin1β (PAP); Sos, son of sevenless (Ras guanyl nucleotide exchange factor (GEF)); RA-GEF-1/2/PDZ-GEF (Rap1GEF); Epac1 (RapGEF); DOCK, dedicator of cytokinesis (RacGEF); RasGAP, Ras GTPase-activating protein; NF1, neurofibromatosis type-1 (RasGAP); RacGDI, Rac guanosine dissociation inhibitor; AGAP (ADP-ribosylation factor (Arf) 1 GAP); ASAP1 (Arf1GAP); ACAP1/2 (Arf6GAP1/2); RA-RhoGAP, Rap-activated RhoGAP; RGS, regulator of G-protein signaling protein; Sin1, SAPK-interacting protein 1 (suppressor of Ras signaling); Arf, ADP-ribosylation factor; Rac1, Ras-related C3 botulinum toxin substrate 1 (Rho family, small GTP binding protein); Drp1, dynamin-related protein 1 (dynamin superfamily GTPase); PDE, cAMP phosphodiesterase; Praja-1 (E3 ubiquitin ligase acting on serotonin transporter); p47*^phox^* (component of NADPH oxidase); α-synuclein (associated with Parkinson’s disease); NF2 (Hippo upstream component); seipin (role in lipid droplet formation); CIDEA, cell-death-induced DFF45-like effector A (lipid droplet protein); Bazooka/Par-3 (cell polarity regulator); Dvl-2, dishevelled homolog (mediator of the Wnt signaling pathway); RPK118 (SphK1-binding protein); Neurogranin (calmodulin-binding protein); β-COP (coatmer protein); NSF, N-ethylmaleimide-sensitive factor (ATPase associated with diverse cellular activity (AAA)); Kinesin (motor protein); Anc, adenine nucleotide carrier protein; Nir (PI-transfer protein); IQGAP1, IQ motif-containing guanosine triphosphatase-activating protein 1 (scaffold protein); CIN85, Cbl-interacting protein of 85 kDa (adaptor/scaffold protein); syntaxin1A (soluble NSF attachment protein receptor (SNARE) protein); CgA, chromogranin A (a representative constituent of the core aggregate within secretory granules); LKB1, liver kinase B1 (serine/threonine protein kinase); TREK-1, TWIK-related K^+^ channel type 1 (potassium channel); Pah1 (PAP); Opi1p (transcriptional repressor); Spo20p (SNARE protein); Sso1p (SNARE protein); Sec18p/NSF (AAA); Ups1 (mitochondrial fusion protein in the inner membrane); Chm7 (part of an ESCRT-III-like complex); ABI1, ABA-insensitive 1 (protein phosphatase); PP2CA, protein phosphatase 2CA; TGD, trigalactosyldiacylglycerol (chloroplast lipid transport protein); AtPDK1 (*Arabidopsis thaliana* 3-phosphoinositide-dependent protein kinase-1); MKK7/9 (mitogen-activated protein kinase kinase 7/9); SnRK2.4, sucrose nonfermenting-1-related protein kinase 2.4; PID, protein kinase PINOID (regulator of auxin signaling); AtSphK1, *Arabidopsis thaliana* sphingosine kinase 1; PEPC, phosphoenolpyruvate carboxylase; LHY (late elongated hypocotyl, transcription factor involved in the circadian clock); CCA1 (circadian clock associated 1, transcription factor involved in the circadian clock); Werewolf (MYB transcription factor); AHL4, AT-hook motif nuclear localized protein 4 (transcription factor to regulate triacylglycerol degradation for seeding establishment); AKT2, potassium channel; MAP65-1, microtubule-associated protein 65-1; RbohD160, respiratory burst oxidase homolog D 160; 14-3-3 protein (member of a family of regulatory molecules); MtDef4, *Medicago truncatula* defensin 4; NsD7, *Nicotiana suaveolens* defensin 7; SNX, sorting nexin (suppressing vascular degradation).

Diacylglycerol kinase (DGK) phosphorylates diacylglycerol (DG) to produce phosphatidic acid (PA) (Figure 1) [1,2,3,4,5]. Both DG and PA are well-established second messengers. Therefore, because DGK can potentially serve as a DG consumer as well as a PA generator (Figure 1), DGK regulates the balance between DG and PA. DG plays important roles in regulating several signal transduction proteins [6,7,8,9], such as conventional protein kinase C (cPKC), novel PKC (nPKC), protein kinase D (PKD, atypical PKC (aPKC)), Ras guanyl nucleotide-releasing protein (GRP), Unc-13 (Uncoordinated-13), chimaerin (Rac-specific GTPase-activating protein (GAP)) and transient receptor potential channel (TRPC, Ca^2+^-permeable cation channel) 2, all of which, except for TRPC2, contain one or two DG-binding C1 domain(s) (Figure 1) [6,9,10].

In addition to DG, PA has been reported to control a number of signaling proteins in mammals [11,12,13,14,15,16,17] such as protein kinases, including Raf-1 (C-Raf) kinase [18,19,20], PKCε (nPKC) [21,22], PKCζ (aPKC) [23] and mammalian target of rapamycin (mTOR) [24]; lipid kinases including phosphatidylinositol (PI)-4-phosphate 5-kinase (PIP5K) [25,26] and sphingosine kinase (SphK) 1 [27]; protein phosphatases including protein phosphatase-1 catalytic subunit (PP1c) [28]; lipid phosphatases including Lipin 1β [29]; phospholipases including phospholipase C (PLC) β1 and γ1 [30]; G-protein regulators including RasGAP [31] and chimaerin [32,33]; G-proteins including ADP-ribosylation factor (Arf) 1 [34] and Rac1 [35,36]; phosphodiesterases including cAMP phosphodiesterase (PDE)-4A1 [37,38] and 4B1 [39]; and miscellaneous proteins including Praja-1 [40], p47*^phox^* [41] and α-synuclein [42,43] (Figure 1). Other than in mammals, many regulatory proteins in insects, yeasts, and plants also associate with PA. It is noteworthy that the number of PA-binding proteins (PABPs) is far greater than that of DG-binding proteins (DGBPs). The greater number may reflect that there are no common binding motifs like the C1 domain for DG binding.

**Figure 2:**
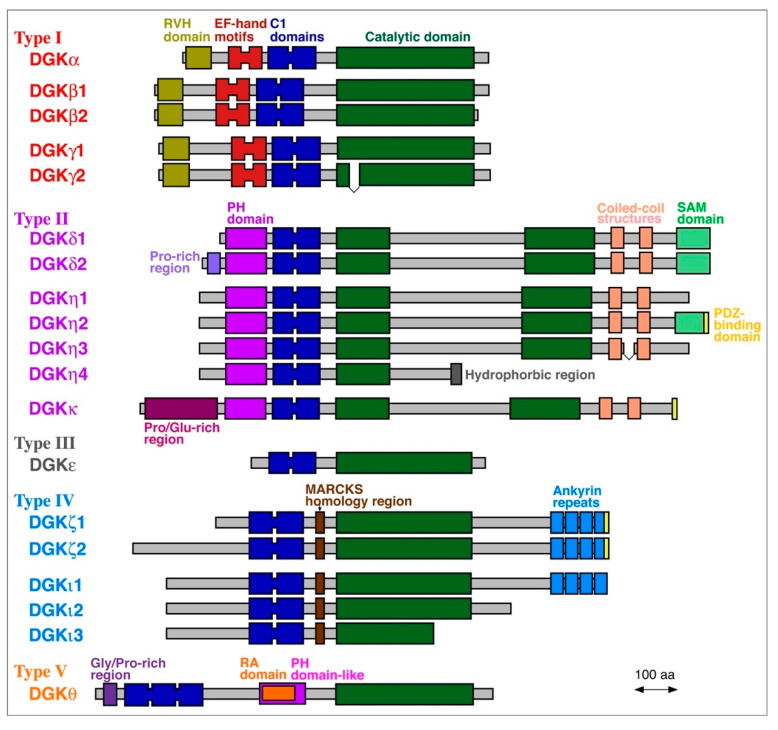
Mammalian DGK family proteins. Alternative splice variants are also shown. MARCKS, myristoylated alanine-rich C-kinase substrate; PDZ, postsynaptic density 95, discs large, zonula occludens-1; PH: pleckstrin homology; RA, Ras-associated; RVH, recoverin homology; SAM, sterile α-motif.

To date, 10 mammalian DGK isozymes (DGKα, β, γ, δ, ε, ζ, η, θ, ι and κ) have been identified (Figure 2) [1,2,3,4,5]. These DGK isozymes commonly contain a catalytic domain and at least two C1 domains (cysteine-rich domains) and are divided into five groups (type I–V) according to their structural features (Figure 2) [1,2,3,4,5]. DGKβ, γ, δ, η, ζ, and ι have one to three alternative splice variants, which add further diversity and complexity to DGK (Figure 2). DGK isozymes have been reported to be involved in many physiological events, including cell proliferation and migration, glucose intake, immunity and neuronal network construction, and pathogenesis of a wide variety of diseases, exemplified by cancer, epilepsy, obsessive-compulsive disorder (OCD), bipolar disorder (BPD), fragile X syndrome (FXS), immunodeficiency, cardiac hypertrophy, hypertension, and type 2 diabetes (T2D) [1,2,3,4,5,44,45,46,47,48].

There are several target proteins that have been strongly suggested to be regulated by DGK isoforms through consumption of DG or production of PA. For DG consumption, it was reported that DGKα [49,50], DGKβ [51] and DGKγ [52] inhibited RasGRP1, PKCβI, and PKCγ, respectively, through consumption of DG (Figure 1). Moreover, type I DGKs (α, β and γ) inhibit TRPC2 via DG reduction [53]. DGKδ interacts with PKCα, PKCδ, PKCε, and PKCη and attenuates their DG-dependent activities [54,55,56]. DGKζ reduces the activities of PKCα [57,58] and RasGRP1 [59]. DGKι inhibits RasGRP3, which activate Rap1 [60]. R59022 (a broad DGK inhibitor)-sensitive DGK isozyme(s) regulates subcellular localization of PKCε via an increase in DG levels [22].

With respect to PA production by DGK, DGKζ was demonstrated to enhance the activities of mTOR [61] and PIP5KIα [62] through increases in PA levels (Figure 1). It was reported that DGKα and DGKγ activated PKCζ [63,64] and β2-chimaerin [65], respectively, probably via PA production. DGKδ was recently revealed to activate Praja-1, the E3 ubiquitin ligase acting on the serotonin transporter in the brain, through PA production [40,66]. It was demonstrated that creatine kinase-muscle type (CKM) functionally associated with DGKδ and was activated by PA [67,68].

**Figure 3:**
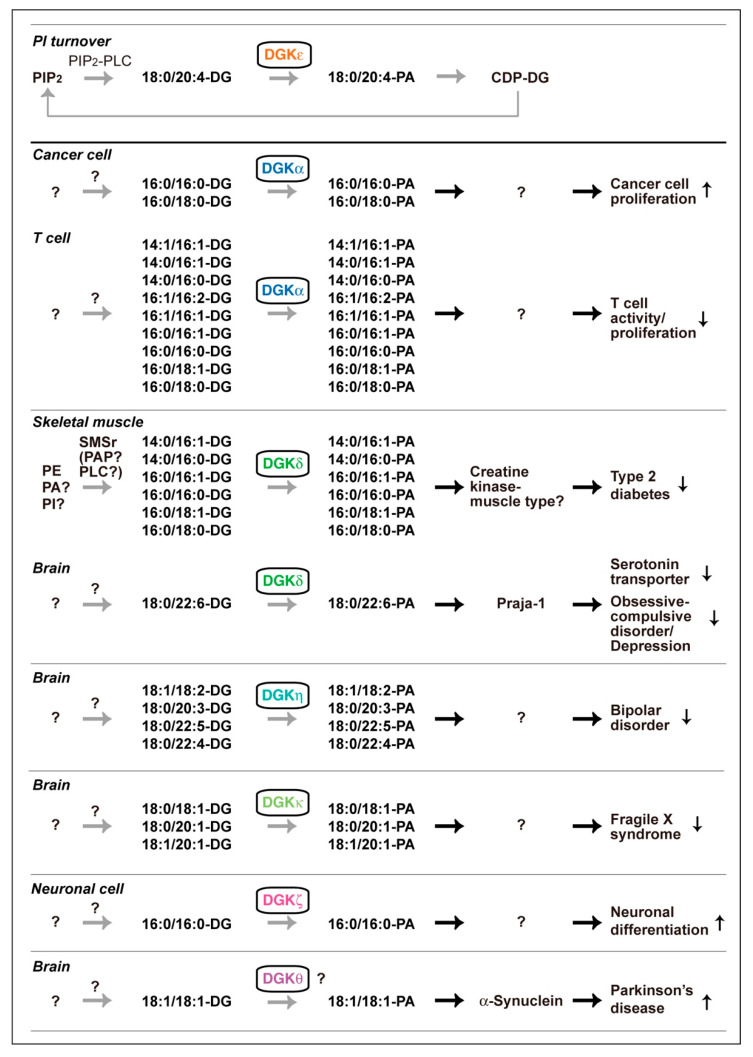
Various DGK isozyme-dependent DG species phosphorylation pathways that are independent of PI turnover. PIP_2_, phosphatidylinositol 4,5-bisphosphate; CDP-DG, cytidine diphosphate diacylglycerol; SMSr, sphingomyelin synthase-related proteins; PAP, PA phosphatase. PA molecular species produced by DGKβ, DGKγ and DGKι have not been determined.

DG and PA consist of a variety of molecular species that have different acyl chains, which have diverse numbers of carbon atoms (14–22) and double bonds (0–6), at the *sn*-1 and *sn*-2 positions; consequently, mammalian cells contain at least 50 structurally distinct DG and PA species. DGK activity is one of the components of the PI turnover. Therefore, it has generally been thought, dogmatically, that DG utilized by DGK is solely derived from phosphatidylinositol (PI) turnover, which exclusively produces 1-stearoyl-2-arachidonoyl-DG (18:0/20:4-DG) (*X:Y* = the total number of carbon atoms: the total number of double bonds in the fatty acyl moiety of the glycerol backbone) (Figure 1). Only DGKε exhibits selectivity to 18:0/20:4-DG in vitro [69,70] and in vivo [71]. However, other isozymes do not have such selectivity in vitro [72,73,74,75,76]. Studies in the recent decade provided data strongly suggesting that DGK isozymes, except for DGKε, utilize a variety of DG species but not PI turnover-derived 18:0/20:4-DG (Figure 3).

As described above, a number of PABPs in mammals have been identified to date (Figure 1) [11,12,13,14,15,16,17]. However, only a few PA species-selective ones have been found. Moreover, the PA species-selectivity of only a small part of PABPs has been determined. Therefore, we attempted to identify PA species-selective binding proteins using 16:0/16:0-PA and 18:0/22:6-PA and, consequently, found several PABPs that interact with different PA species.

In this review, we shed light on PAs, especially the diversity of PA molecular species, produced by DGK isozymes and on PABPs, especially those that possess selectivity for PA molecular species. We will also touch on a PI turnover-independent upstream pathway of DGK that was recently found. Moreover, a new PA probe, which is reliable and widely applicable, will be briefly mentioned.

## 2. PA Molecular Species Produced by DGK Isozymes Except for DGKε

DGKε (type III) was purified and it clearly showed selectivity for 18:0/20:4-DG in vitro (Figure 3) [70]. Moreover, cDNA-cloned DGKε selectively phosphorylated 18:0/20:4-DG in vitro [69]. Rodriguez de Turco et al. reported that knockout (KO) of DGKε indeed disturbed PI turnover [71]. Interestingly, DGKε is strongly expressed in Purkinje cells of the cerebellum and pyramidal cells of the hippocampus and regulates seizure susceptibility and long-term potentiation [71], which are governed by PI turnover. Taken together, these results indicate that DGKε is an essential component of PI turnover and exerts its physiological functions as a component of PI turnover in the brain (Figure 3).

On the other hand, nine other isozymes, except for DGKε, fail to show selectivity for 18:0/20:4-DG in vitro [72,73,74,75,76]. Therefore, we questioned whether these nine isozymes indeed utilize PI turnover-derived 18:0/20:4-DG species in cells and organs. However, it has been difficult to quantitatively determine small changes in PA species levels caused by KO and silencing of a DGK isozyme in physiological and pathological events. Liquid chromatography-mass spectrometry (LC-MS) is a powerful tool to detect different molecular species of phospholipids in cells. However, PA was difficult to detect with high accuracy and reproducibility in the general LC conditions because of ion suppression by other major phospholipids, phosphatidylcholine (PC) and sphingomyelin [77]. Therefore, we optimized mobile phases using silica column LC to separate PA from major phospholipids and confirmed that PA species were quantitatively and reproducibly detected in LC-MS [77]. Then, we determined the PA molecular species produced by DGK isozymes in cells and organs and found that a variety of PA species other than 18:0/20:4-PA were generated by DGK isozymes. Intriguingly, these results do not support the dogma that all DGK isozymes utilize DG derived from PI turnover and instead support a new view that DGK isozymes, except for DGKε, utilize DG species derived from pathways independent of PI turnover, as described below (Figure 3).

### 2.1. DGKα

DGKα, which is a type I isozyme, was first identified by cDNA cloning [78,79]. This isozyme possesses a recoverin homology (RVH) domain and tandem repeats of two Ca^2+^-binding EF-hand motifs [80,81] (Figure 2). Several lines of evidence suggested that the Ca^2+^-induced dissociation of the intramolecular interaction between the EF-hand motifs and the C1 domains of DGKα is the key event that regulates the activity of the enzyme [82,83,84].

DGKα has been repeatedly reported to be a promising target for anticancer drugs [47,85]. DGKα, which is abundantly expressed in several cancer cells, such as melanoma [63,86], hepatocellular carcinoma [87], and lymphoma [88], enhances cell proliferation and inhibits apoptosis. Moreover, it has been noted that DGKα activates angiogenesis signaling [89] and that this isozyme plays a key role in cancer cell migration [90]. Recently, LC-MS revealed that the production of palmitic acid (16:0)-containing PA species such as 16:0/16:0- and 16:0/18:0-PA were attenuated by CU-3, a DGKα-selective inhibitor [91], in AKI melanoma cells under starved conditions (Figure 3) [92]. Therefore, these findings support the insight that inhibitory analogs (antagonists) of 16:0/16:0- and 16:0/18:0-PA can be therapeutics against tumor cell growth.

In addition to being expressed in cancer cells, DGKα is highly expressed in T cells [78]. In contrast to cancer cells, DGKα facilitates the immune nonresponsive (nonproliferation) state known as T cell clonal anergy [49,93,94]. T cell anergy induction represents the main mechanism by which advanced tumors avoid immune action [95]. Indeed, DGKα limits the antitumor immune response by tumor-infiltrating CD8^+^ T cells [96]. Therefore, the inhibition of DGKα activity is thought to enhance T cell activity, which governs cancer immunity [44,85,97,98]. We recently found that palmitic acid (16:0)- and/or palmitoleic acid (16:1)-containing phosphatidic acids such as 14:1/16:1-, 14:0/16:1-, 14:0/16:0-, 16:1/16:2-, 16:1/16:1-, 16:0/16:1-, 16:0/16:0-, 16:0/18:1- and 16:0/18:0-PA are generated by DGKα in starved Jurkat T cells (Figure 3) [99]. Intriguingly, the profile in starved T cells (palmitic acid- and/or palmitoleic acid (16:1)-containing PA species) [99] is different from that in starved melanoma cells (palmitic acid-containing PA species) [92]. Therefore, DGKα generates distinct PA species in different cells, and the differences in the PA molecular species may account for the opposing functions of DGKα in cancer and T cells.

DGKα-selective inhibitors would be dual effective compounds (i.e., ideal cancer therapy candidates) because, as described above, they attenuate cancer cell proliferation and simultaneously enhance immune responses, including anticancer immunity [100]. Indeed, a DGKα-selective inhibitor, CU-3, induced both cancer cell apoptosis and T-cell activation [91,92]. Other DGKα-selective inhibitors, ritanserin [101] and analogs of Amb639752 (11 and 20) [102], were recently reported as well. These compounds are expected to be able to become ideal cancer drugs.

### 2.2. DGKζ

DGKζ (type IV) contains a MARCKS (myristoylated alanine-rich C kinase substrate) phosphorylation site domain and four ankyrin repeats (Figure 2) [72,103]. Topham et al. [104] demonstrated that the nuclear-localization signal of DGKζ is located in a MARCKS phosphorylation site domain and that PKCs α and γ regulate the mode of DGKζ localization by phosphorylation of the domain. DGKζ-mediated synaptic conversion of DG to PA is required for the maintenance of dendritic spines [105]. Moreover, DGKζ, syntrophin, and Rac1 form a ternary signaling complex that controls neurite outgrowth in N1E-115 neuroblastoma cells [106].

Previous reports showed that the level of PA was increased during neuronal differentiation [107,108]. However, it has not been revealed what PA molecular species are produced. Recently, 16:0/16:0-PA and, to a lesser extent, 14:0/16:0-PA and 16:0/18:0-PA, were found to be exclusively generated during differentiation of Neuro 2A neuronal cells in a DGKζ-dependent manner (Figure 3) [109].

DGKζ1 (Figure 2), but not DGKζ2, was physically associated with RasGRP1 and attenuated RasGRP1 activity by DG consumption [59]. Therefore, in addition to DGKα, DGKζ acts as a suppressor of T cell functions and its inhibitors are expected to be useful for cancer immunotherapy [44,85,98,110]. DGKs ζ and α appear to share the same function (inhibition of RasGRP1 activity in T cells and consequently attenuation of T cell activity). Indeed, the combination of the inhibition of DGKα and DGKζ additively or synergistically induces activation of T cells [111]. However, it is still unclear what PA species are generated by DGKζ in T cells.

### 2.3. DGKδ

DGKδ [112,113] has a pleckstrin homology (PH) domain at its N-terminus and a sterile α motif (SAM) domain at its C-terminus (Figure 2). Alternative splice variants, δ1 and δ2, have different N-terminal regions (Figure 2) [112,113]. cPKC phosphorylates the PH domain of DGKδ and regulates its subcellular localization [114]. The isozyme forms homo-oligomers via its SAM domain [115,116,117].

DGKδ is strongly expressed in the skeletal muscle [112]. DGKδ regulates glucose transport [54,56,118] and contributes to exacerbating the severity of type 2 diabetes (T2D) [54,56]. It was recently found that, in response to high glucose-stimulation, 16:0- and/or 16:1-containing PA species such as 14:0/16:1-PA, 14:0/16:0-PA, 16:0/16:1-PA, 16:0/16:0-PA, 16:0/18:1-PA, and 16:0/18:0-PA were generated by DGKδ in C2C12 myoblast cells (Figure 3) [76].

Interestingly, we recently demonstrated that myristic acid (14:0) increased the expression of DGKδ and enhanced glucose uptake in C2C12 myotube cells [118,119]. Moreover, chronic oral administration of myristic acid improved hyperglycaemia by decreasing insulin-responsive glucose level in Nagoya-Shibata-Yasuda mice, a spontaneous model for studies of obese T2D [120]. These results indicate that myristic acid is a potential candidate for the prevention and therapy of T2D and its related diseases.

DGKδ is also highly enriched in the brain [121]. Recently, we generated and analyzed brain-specific DGKδ-KO mice and found that the KO mice show a selective serotonin reuptake (serotonin transporter (SERT)) inhibitor (fluoxetine)-sensitive OCD-like behaviors [122]. Moreover, the DGKδ-deficiency increased the amount of SERT protein in the mouse cerebral cortex [123]. DGKδ interacted with SERT [40,123], melanoma antigen gene-D1 (MAGE-D1) [40], and Praja-1 E3 ubiquitin-protein ligase [40], which ubiquitinates SERT [124], and induced SERT degradation in a DGK activity-dependent manner [40]. It is noteworthy that only the level of 1-stearoyl-2-docosahexaenoyl (18:0/22:6)-PA was decreased in the DGKδ-KO mouse brain [66], suggesting that DGKδ generates 18:0/22:6-PA in the brain. Intriguingly, 18:0/22:6-PA selectively bound to Praja-1 and enhanced its activity (see Table 1) [66]. These results indicate that DGKδ generates distinct PA species in different tissue/cells and/or in response to different stimuli.

It is known that docosahexaenoic acid (DHA, 22:6, ω-3) deficiency occurs during aging and dementia and that the deficiency impairs memory and learning, exacerbates anxiety and depression, and promotes age-related neurodegenerative diseases, including Alzheimer’s disease [214]. DHA is asserted to increase membrane fluidity, strengthen antioxidant activity, and plays anti-inflammatory roles [214]. However, all these effects chemically/physically, nonselectively, and indirectly affect the brain functions. On the other hand, DHA-containing PA biologically, selectively, and directly activates Praja-1 E3 ubiquitin-protein ligase and, consequently, reduces the amount of SERT protein [215], which attenuates the serotonergic system and is the target of anti-depression and anti-OCD drugs [216,217]. Therefore, it is possible that DHA incorporated into PA (and chemical compounds mimicking 18:0/22:6-PA) biologically, selectively, directly, and most effectively protect the brain dysfunctions listed above.

### 2.4. DGKη

Like DGKδ, DGKη (type II) possesses a PH domain and a SAM domain (only DGKη2) (Figure 2) [218,219]. The PH domain of DGKη interacts with PIP_2_ [220]. DGKη forms homo-oligomers and hetero-oligomers with DGKδ via their SAM domains [219]. DGKη is required for the Ras–B-Raf/C-Raf–mitogen-activated protein kinase/ERK kinase (MEK)–extracellular signal-regulated kinase (ERK) signaling cascade in cancer-derived cells [221].

Successive genome-wide association studies (GWASs) indicated a possible relationship between single nucleotide polymorphisms (rs9315885, rs1012053 and rs1170191) of *DGKH* (DGKη gene) and BPD [222,223,224,225,226]. Indeed, DGKη-KO mice exhibited BPD mania-like phenotypes [227]. Moreover, microarray analysis showed that mRNA levels of prolactin and growth hormone, which are augmented in BPD patients and BPD model animals, are most strongly increased [228]. Furthermore, it was revealed that the amount of dopamine is augmented in the DGKη-deficient mouse brain [Asami, M. and Sakane F. unpublished work]. The levels of polyunsaturated fatty acid (PUFA)-containing PA species such as 18:1/18:2-, 18:0/20:3-, 18:0/22:5-, 18:0/22:4-, and 18:0/22:3-PA were recently found to be decreased in the DGKη-KO brain (Figure 3) [228], suggesting that DGKη generates these PUFA-containing PA species in the brain.

### 2.5. DGKκ

DGKκ, which is a type II isozyme, possesses a PH domain at the N-terminus (Figure 2) [229]. DGKκ, but not other type II DGKs, is tyrosine-phosphorylated at Tyr-78 in the N-terminal, κ-isoform-specific extension through the Src family kinase pathway in response to oxidative stress [229]. Moreover, the stress inhibits DGKκ activity.

It is worthy of note that Moine’s group recently generated DGKκ-KO mouse and revealed the relationship between DGKκ and FXS [230]. FXS is caused by abnormal CGG-repeat expansion at the FMR1 gene, which codes the RNA-binding protein, fragile X mental retardation protein (FMRP). Interestingly, FMRP was found to increase DGKκ activity in neurons. In DGKκ-deficient cortical neurons, L-quisqualic acid (a group 1 metabotropic glutamate receptor agonist)-dependent increases in 36:1 (18:0/18:1)-, 38:1 (18:0/20:1)-, and 38:2 (18:1/20:1)-PA levels were attenuated (Figure 3).

### 2.6. DGKθ

DGKθ (type V) has three C1 domains, a glycine/proline-rich region, a Ras association domain and a PH domain-like region (Figure 2) [74]. GWAS suggested that single nucleotide polymorphisms (rs1564282 and rs11248060) of *DGKQ* (DGKθ gene) are associated with a higher risk of Parkinson’s Disease [231,232]. DGKθ is highly expressed in the cerebellum and hippocampus in the adult rat brain [74]. Intriguingly, overexpression of DGKθ mainly increases the amount of 18:1/18:1-PA in mouse primary hepatocytes (Figure 3) [233]. It is interesting that the PA species strongly binds to α-synuclein (see Table 1), which is associated with the pathogenesis of Parkinson’s Disease (see Section 3.1) [42].

## 3. Molecular Species Selectivity of PABP

A number of proteins such as protein kinases, lipid kinases, protein phosphatases, lipid phosphatases, phospholipases, G-proteins, G-protein regulators, and phosphodiesterases have been identified as PABPs to date (Table 1) [11,12,13,14,15,16,17].

34:1 (16:0/18:1)-PA is generally abundant in mammalian cells, tissues and organs. For example, in the mouse brain, the abundance of PA species is in the order of 34:1 (16:0/18:1)-PA = 36:1 (18:0/18:1)-PA, 36:2 (18:1/18:1)-PA = 38:4 (18:0/20:4)-PA and 38:1 (18:0/20:1)-PA [228]. In mouse myoblast cells, 34:1 (16:0/18:1)-PA is most abundant, followed by 36:2 (18:1/18:1)-PA, 34:2 (16:1/18:1)-PA, 36:1 (18:0/18:1)-PA, and 32:1 (14:0/16:1)-PA [76]. Therefore, in many cases, the screening for detecting PABPs was performed with a major PA species, 16:0/18:1-PA. Moreover, because the molecular species selectivity of PABPs has not attracted attention so far, PA species mixtures were used to detect PABPs in many cases. However, as described previously, we recently found that several DGK isozymes generate diverse PA species. It is possible that the general screening with 16:0/18:1-PA and PA mixtures missed some PABPs that are selective for minor PA species. Therefore, we recently started a comprehensive screening for PABPs in the skeletal muscle and brain using several minor PA species, including 16:0/16:0-PA, which is generated by DGKδ in myoblast cells [76] and DGKζ in neuronal cells [109], and 18:0/22:6-PA, which is produced by DGKδ in the brain [66]. As a result, we found several new PABPs that have different selectivities to PA species. In addition to PA-selective PABPs discovered by us, there are only several such PABPs. Intriguingly, these PABPs do not exhibit the selectivity to 18:0/20:4-PA, which is derived from the PI turnover, indicating that they interact with PI turnover-independent PA species.

### 3.1. α-Synuclein

α-Synuclein has been implicated in Parkinson’s Disease [234] because this protein is the main constituent of Lewy bodies in patients with the disease as well as in the bodies of patients suffering from dementia [235]. We screened 16:0/16:0-PA-binding proteins from the mouse brain and, consequently, identified α-synuclein [42]. α-Synuclein was already reported to be an acidic phospholipid (phosphatidylserine (PS) and PA)-binding protein [236]. However, the binding assay between α-synuclein and acidic phospholipids has commonly employed only 16:0/18:1-PA [237,238,239] because this species is the major species in the brain [228,240]. When we determined the binding affinities of several PA species, 18:1/18:1-PA was found to much more intensely bind to α-synuclein than 18:1/18:1-PS and 16:0/18:1-PA (Table 1) [42]. The binding intensity of 18:1/18:1-PA was also stronger than that of 16:0/16:0-PA, 18:0/18:0-PA, and 18:0/20:4-PA (Table 1). Moreover, 18:1/18:1-PA markedly induced secondary structural changes (increased α-helix content) and aggregation formation of α-synuclein. Therefore, 18:1/18:1-PA is likely to be the strongest binding partner of α-synuclein among the phospholipids examined so far. Inhibitory analogs (antagonists) of 18:1/18:1-PA may slow down the progression of Parkinson’s Disease via preventing aggregation formation of α-synuclein.

As described previously, DGKθ, which has been reported to be associated with the risk of Parkinson’s Disease [231,232], preferentially produced 18:1/18:1-PA [233]. Interestingly, the content of PA increased in aged male mice (12–14 months old), but that of PS decreased with age [240]. Aging is the greatest risk factor for developing sporadic Parkinson’s Disease [241]. Moreover, Parkinson’s Disease incidence is 1.5 times higher in men than women [242,243]. Therefore, it is possible that 18:1/18:1-PA produced by DGKθ enhances the pathogenesis of Parkinson’s Disease (Figure 3).

### 3.2. Praja-1

As described previously (Section 2.3), Praja-1 E3 ubiquitin-protein ligase [40] interacts with MAGE-D1, an adaptor protein for ubiquitin-dependent degradation [244] and DGKδ, and ubiquitinates SERT [124]. Ubiquitinated SERT is quickly degraded in proteasomes and, consequently, the serotonergic system, especially the level of serotonin in the synaptic cleft, is upregulated. Praja-1 was recently found to strongly interact with 18:0/22:6-PA but not 16:0/16:0-PA, 16:0/18:1-PA, 18:1/18:1-PA, 18:0/20:4-PA, 18:0/18:0-PA or PS [66] (Table 1). Moreover, it is noteworthy that the E3 ubiquitin-protein ligase activity of Praja-1 is selectively enhanced by the DHA-containing PA, 18:0/22:6-PA, which is generated by DGKδ in the brain [66].

### 3.3. Synaptojanin-1

We screened for 18:0/22:6-PA-binding proteins in the mouse brain. As a result, synaptojanin-1 was identified [Hoshino, F. and Sakane, F., unpublished work]. Synaptojanin-1 dephosphorylates the D-5 position phosphates from PI(4,5)P_2_ [245] and is a key player in the clathrin-mediated synaptic vesicle cycle [246]. However, it is interesting that synaptojanin-1 intensely binds to PUFA-containing-PAs, 18:0/20:4-PA and 18:0/22:6-PA (Table 1). However, the protein did not show strong binding activities for 16:0/16:0-PA, 16:0/18:1-PA, 18:1/18:1-PA, 18:0/18:0-PA, or another anionic phospholipid 18:0/22:6-PG. Therefore, it is likely that synaptojanin-1 is an 18:0/20:4-PA- and 18:0/22:6-PA-selective binding protein but not a nonselective anionic phospholipid-binding protein.

### 3.4. L-Lactate Dehydrogenase (LDH) A

LDHA in skeletal muscle is an energy-metabolizing enzyme critical for tumor-related anaerobic respiration [247]. LDHA was already reported to bind to acidic phospholipids such as PS and cardiolipin (CL), at acidic pH [248]. However, at physiological pH (7.4), 18:0/18:0-PA, 18:0/20:4-PA, and 18:0/22:6-PA more strongly interact with LDHA (Table 1) than PS or 16:0/16:0-PA [68]. Moreover, PUFA-containing PAs, 18:0/20:4-PA and 18:0/22:6-PA, but not a saturated fatty acid (SFA)-containing PA, 18:0/18:0-PA, induced secondary structural changes (decreased α-helix content) of LDHA and attenuated its activity.

It was reported that LDHA is upregulated in human tumors, including glioblastoma [249,250,251]. The Warburg effect, which is the anaerobic metabolism by tumor cells even under well-oxygenated conditions, has been suggested to be an adaptive mechanism to maintain the biosynthetic requirements of uncontrolled proliferation [252]. LDHA is a key enzyme of the Warburg effect [247,253,254]. Indeed, silencing/genetic disruption of LDHA inhibited tumor growth in vitro and in vivo [255,256,257]. It is noteworthy that arachidonic acid (20:4)- and DHA (22:6)-containing DG were decreased within tumor regions [258]. Therefore, it is likely that a decrease of the PA molecular species containing PUFA, arachidonic acid or DHA, cannot attenuate the activity of LDHA in tumor cells. It is possible that chemical compounds that mimic 18:0/20:4-PA and 18:0/22:6-PA can be drugs against tumor cell growth.

### 3.5. CKM

CKM is also an energy metabolizing enzyme and has long been known to be correlated with T2D [259,260,261,262]. We recently identified CKM by screening using 16:0/16:0-PA liposomes and found that SFA and/or monounsaturated fatty acid (MUFA)-containing-PA species (16:0/16:0-PA, 16:0/18:1-PA, 18:1/18:1-PA and 18:0/18:0-PA) but not PUFA-containing PAs (18:0/20:4-PA or 18:0/22:6-PA) were associated with CKM [67] (Table 1). Moreover, 16:0/16:0-PA, 16:0/18:1-PA, 18:1/18:1-PA, and 18:0/18:0-PA enhanced CKM activity [68]. CKM and DGKδ coexpressed in COS-7 cells were well colocalized with each other depending on DGKδ activity [Hoshino F. and Sakane F. unpublished work]. Therefore, it is possible that a decrease in SFA/MUFA-PA species caused by the attenuated expression of DGKδ in the skeletal muscle of T2D patients [54] adversely affects the localization and activity of CKM and leads to energy metabolic failure, exacerbating T2D. It is possible that chemical compounds that mimic SFA/MUFA-PA species such as 16:0/16:0-PA, 16:0/18:1-PA, 18:1/18:1-PA, and 18:0/18:0-PA suppress the pathogenesis of T2D.

### 3.6. DGKγ

DGKγ acts as a suppressor of Rac1-lamellipodium formation [263]. Shirai’s group demonstrated, using DGKγ-KO mice, that this isozyme regulates cerebellar motor coordination, long-term depression, and the dendritic development of Purkinje cells [264]. Interestingly, DGKγ, a PA-producing enzyme, recursively associates with PA [141]. However, other DGK isozymes (DGK α, β, δ, η, κ, ε, ζ, ι and θ) failed to show such PA-binding activities. Although only protein-lipid overlay assays were performed, 18:1/18:1-PA and 18:0/20:4-PA (MUFA- or PUFA-containing PAs) more intensely bound to DGKγ than 14:0/14:0-PA or 18:0/18:0-PA (SFA alone-containing PA) (Table 1) [141].

### 3.7. Raf-1 (C-Raf)

Raf-1 (C-Raf) kinase is a serine/threonine protein kinase and is related to a retroviral oncogene. Raf-1 is a component of the Ras–Raf–MEK–ERK signal transduction pathway, which is involved in various growth factor-induced cell responses such as cell division. Although the effects of only three PA species were determined, Raf-1 was found to more strongly bind to MUFA-containing PAs (16:1/18:1-PA and 18:1/18:1-PA) than a SFA alone-containing PA (16:0/16:0-PA) (Table 1) [18,19,20].

### 3.8. mTORC2

mTORC2, which is a rapamycin-insensitive protein complex 2 containing serine/threonine kinase mTOR, regulates cell proliferation/survival and cell migration. Although only three PA species were examined, 16:0/16:0-PA, a SFA-containing PA, much more strongly bound to mTORC2 than 16:0/18:1-PA and 18:1/18:1-PA, SFA- and/or MUFA-containing PAs (Table 1) [131].

### 3.9. PDE4A1 and A5

PDE4A1 and PDE4A5 are cAMP-specific hydrolyzing family members, which regulate cAMP-dependent signaling cascades. PDE4A1 binds more intensely to SFA-, MUFA- and/or PUFA-containing PAs, 16:0/18:1-PA, 16:0/18:2-PA, 18:1/18:1-PA, and 18:0/18:1-PA, than 18:0/22:6-PA, a DHA-containing PA (Table 1) [37,38]. PDE4A5 interacts more strongly with SFA alone-containing PAs, 16:0/16:0-PA and 18:1/18:1-PA, than MUFA- or PUFA-containing PAs, 16:0/18:1-PA, 18:0/18:1-PA, 18:0/20:4-PA and 18:0/22:6-PA (Table 1) [166].

### 3.10. Seipin

Seipin, which is an integral membrane protein in the ER, is important for lipid droplet formation. Although only a few PA species were tested, 16:0/18:1-PA, a SFA- and MUFA-containing PA, more strongly associated with seipin than 16:0/16:0-PA or 18:0/18:0-PA, SFA alone-containing PAs (Table 1) [169].

### 3.11. Plant PABPs

The PA species selectivity of plant PABPs has been well analyzed compared with mammalian PABPs. Several plant PABPs exhibit their unique PA species selectivity (Table 1). ABI1 (ABA-insensitive 1, protein phosphatase) prefers 18:1/18:1-PA [193]. LHY (late elongated hypocotyl, transcription factor involved in the circadian clock) [205] and CCA1 (circadian clock associated 1, transcription factor involved in the circadian clock) [205] strongly bind to 16:0/16:0-PA. AHL4 (AT-hook motif nuclear localized protein 4, transcription factor to regulate triacylglycerol degradation for seeding establishment) [207] and MKK7/9 (mitogen-activated protein kinase kinase 7/9) [199] possess high affinity to 16:0/18:1-PA and 18:1/18:1-PA. Potassium channel AKT2 prefers 16:0/18:2-PA [208]. PP2CA (protein phosphatase 2CA, negative modulator of the AKT2 activity) intensely associates with 18:1/18:1-PA and 18:2/18:2-PA [194]. PID (protein kinase PINOID, regulator of auxin signaling) and MAP65-1 (microtubule-associated protein 65-1) preferentially bind to PUFA-containing PA (18:2/18:2-PA) [201]. RbohD160 (respiratory burst oxidase homolog D 160) strongly interacts with MUFA- and PUFA-containing PAs (16:0/18:1-PA, 18:1/18:1-PA and 16:0/18:2-PA) [210].

## 4. PA Probe

As described previously, PAs plays important physiological roles as second messengers. Therefore, tracking the localization and dynamics of intracellular PA is essential for understanding a wide variety of physiological and pathological events regulated by PA. Several PA-binding domains (PABDs), such as Spo20p-PABD [38,186] and PDE4A1-PABD [37,38], are often used as PA probes [265,266,267]. However, they exhibit their own subcellular localization to the plasma membrane (Spo20p-PABD) and Golgi apparatus (PDE4A1-PABD) in a cell stimulation-independent manner (a cell stimulation-induced PA generation-independent manner) [15,38]. The cell stimulation-independent localization disturbs their functions as PA probes and, consequently, makes them relatively difficult to apply. Therefore, a reliable and widely applicable PA probe that can be used for any cell stimulation and cell type has not been sufficiently developed to date.

In this context, α-synuclein N-terminal region (α-synuclein-PABD) is useful for PA sensing in living cells [43]. The region does not exhibit its own subcellular localization to cell membranes such as the plasma membrane and Golgi apparatus in a cell stimulation-independent manner, in contrast to PA sensors developed so far. It was confirmed that α-synuclein-PABD was able to sense physiologically produced, endogenous PA in phagosomes [268]. Moreover, it is interesting to note that the probe detected PA at the peripheral regions (close to the plasma membrane) of neuronal growth cones [268].

α-Synuclein-PABD strongly binds to MUFA-containing PA (18:1/18:1-PA) and only moderately interacts with SFA alone-containing and PUFA-containing PAs (Table 1) [42,43]. Therefore, α-synuclein-PABD cannot detect all PA species in cells. Thus, it is expected that PA probes selective for SFA alone-containing and PUFA-containing PAs will be developed.

## 5. DG-Providing Pathway Upstream of DGK

How do DGK isozymes produce distinct PA species? DGK isozymes, except for DGKε, have no DG species selectivity in vitro, implying that there are different upstream DG supply pathways and/or DG pools, which are independent of PI turnover and provide various DG species to each DGK isozymes. Thus, it is speculated that DG supply pathway(s) upstream of DGK provide certain DG species.

**Figure 4:**
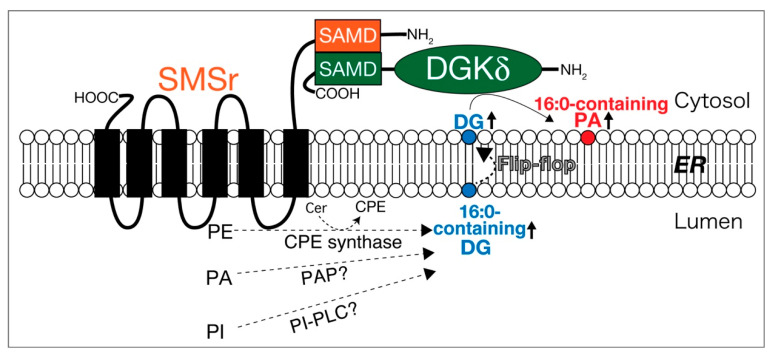
SMSr interacts with DGKδ and supplies DG. SAMD, SAM domain; Cer, ceramide; CPE, ceramide phosphoethanolamine; ER, endoplasmic reticulum.

Sphingomyelin synthase-related protein (SMSr) is a six-transmembrane protein in the endoplasmic reticulum (ER), which generates DG and ceramide phosphoethanolamine (CPE) by utilizing phosphatidylethanolamine (PE) and ceramide (Figure 4) [269]. A SAM domain in SMSr form a homo-oligomer [270,271]. DGKδ also possesses a SAM domain and forms homo-oligomers via the domains [112,113]. Intriguingly, the SAM domains in SMSr and DGKδ are primary structurally similar to each other [270,271]. It is noteworthy that we recently found that SMSr and DGKδ interacted with each other through their SAM domains (Figure 4) [272]. Moreover, overexpression of both SMSr and DGKδ, but not DGKδ or SMSr alone, enhanced PA production in COS-7 cells. In particular, the levels of 16:0- and/or 16:1-containing PA species including 16:1/16:1-PA, 16:0/16:1-PA, 16:0/16:0-PA, 16:1/18:1-PA, and 16:0/18:1-PA, which were also produced by DGKδ in high glucose-stimulated C2C12 myoblast cells [76], were significantly increased. Moreover, SMSr overexpressed in COS-7 cells generated 16:0- and/or 16:1-containing DG species [272]. Taken together, these results strongly suggest that SMSr acts upstream of DGKδ and supplies limited species of DG to DGKδ (Figure 4). Although SMSr produces DG at the lumen side of the ER, DGKδ2 exists in the cytosol [112,113]. However, DG quickly diffuses across the lipid bilayer by the flip-flop mechanism (Figure 4) [273]. Therefore, it is likely that the DG produced by SMSr immediately transverses the ER membrane from the lumen side to the cytosol leaflet and, consequently, is provided to DGKδ (Figure 4).

Puzzlingly, SMSr shows only slight CPE synthase activity [269]. However, it is interesting to note that, in addition to CPE synthase activity, SMSr protein, which was expressed using the baculovirus-insect cell system and highly purified, generated DG through the activities of PA phosphatase (PAP) and PI-PLC in vitro (Figure 4) [Murakami, C. and Sakane, F. unpublished work]. These activities were much stronger than the CPE synthase activity. Moreover, SMSr as PAP prefers SFA and/or MUFA-containing PA species (16:0/16:0-PA and 16:0/18:1-PA) but not PUFA-containing PA species (18:0/20:4-PA or 18:0/22:6-PA). Therefore, these results further support that the supply of DG by SMSr (PAP and PI-PLC) is independent of PI turnover.

Unlike myoblast cells [76], DG species (18:0/22:6-PA) utilized by DGKδ in the brain are not 16:0-containing DG (Figure 3) [66]. In addition to DGKδ, PA species produced by DGKα in melanoma and T cells are also different from each other (Figure 3) [92,99]. The results imply that DGK isozymes utilize distinct DG-supplying pathways in different organs/tissues/cells and/or in response to different cell stimuli. DGKδ was found to interact with SMSr via the SAM domain. However, only DGKδ1, δ2, and η2 have the SAM domain (Figure 2). Thus, other DGK isozymes lacking the SAM domain would utilize other DG-providing pathways instead of SMSr. It is urgently needed to explore other DG supply enzymes/pathways.

## 6. Physiological Implication of Diversity of PA Molecular Species and PABPs

Unlike DGBPs, which have the common DG-binding domain (the C1 domain), obviously common PA-binding motifs, like the C1 domain, have not been identified in PABPs (Table 1). The lack of communality may generate the high diversity of PABPs, which have different selectivity to PA species.

PA is the simplest glycerophospholipid. Hydrophilic head groups of PI (phosphate + inositol ring), CL (phosphate + phosphatidylglycerol (PG)), PG (phosphate + glycerol), PS (phosphate + serine), PE (phosphate + ethanolamine) and PC (phosphate + choline) are considerably larger than PA (phosphate alone). PA forms a cone-like molecular shape, rather than the cylindrical shape typical of other glycerophospholipids [16,274]. The shape of PA likely generates void space surrounding PA molecules. Taken together, it is speculated that PABP can easily access the fatty acid moieties of PA. If this is the case, fatty acid composition of PA would be more physiologically significant than those of other phospholipids. In contrast, DGBPs do not show obvious DG species selectivity, exemplified by PKC [275]. Because DG has only hydroxy group as the hydrophilic head, the lipid is deeply embedded in the lipid bilayer membrane. Thus, DGBPs would have difficulties accessing the fatty acid moieties of DG. However, to prove the hypothesis that PABP can easily access the fatty acid moieties of PA, 3D structures and molecular dynamics simulations of PABPs associated with PA molecule are further needed.

The results recently obtained suggest that DGK–PA–PABP axes can potentially construct a large and complex signaling network. DGK isozymes generate various PA species. Moreover, several PA species-selective PABPs, which regulate their related functions, have been found, and the list of PA species-selective PABPs is still growing. In addition to DGK, phospholipase D (PLD) [276] generates PA as a signaling lipid through the hydrolysis of PC (Figure 1). It has been reported that many PABPs are controlled by PLD-dependent PA [11,14,15,16,17]. Although DGK and PLD commonly generate PA, the profiles of PA species would be distinct from each other. PLD employs only PC as a substrate. On the other hand, for example, DGKδ can utilize DG species derived from PA, PI, and PE through PAP, PI-PLC and CPE synthase activities of SMSr [Murakami, C. and Sakane, F. unpublished work]. Therefore, it is likely that the variation of PA species produced by DGK is higher than that by PLD. Lysophosphatidic acid acyltransferase (LPAAT) also generates various PA species (Figure 1), which are basically utilized as precursors of various phospholipids. Interestingly, there are several LPAAT isozymes that can add different fatty acids to LPA [277,278,279]. PLD- and LPAAT-derived PA species, which can also bind to PA species-selective PABP, together with DGK-derived PA species would confer complexity to the PA molecular species-signaling network. The network consists of various PA producing enzymes including DGK isozymes, various PA molecular species and various PABPs and may regulate a wide variety of physiological functions and pathogenesis.

In mammals, yeasts and plants, different PA species are enriched. As previously described, in mammalian myoblast cells, 34:1 (16:0/18:1)-PA is most abundant, followed by 34:2 (16:1/18:1)-PA = 36:2 (18:1/18:1)-PA, 36:1 (18:0/18:1)-PA and 32:1 (16:0/16:1)-PA [76]. In mammalian brain, PA species are enriched in the order of 34:1 (16:0/18:1)-PA = 36:1 (18:0/18:1)-PA, 36:2 (18:1/18:1)-PA = 38:4 (18:0/20:4)-PA and 38:1 (18:0/20:1)-PA [228]. In contrast, yeast has only C16 and C18 with no or one double bond and is relatively rich in 16:1/16:1-PA and 16:1/18:1-PA [280,281]. PA species in plants are relatively double-bond rich. For example, in the model plants *Arabidopsis thaliana* and soybean, 34:2-PA is most abundant, followed by 34:3-PA, 36:4-PA, and 36:5-PA [282,283]. Therefore, it is likely that these organisms utilize different PA species for their cellular signaling systems and that different PA species construct distinct PA molecular species-signaling networks.

## 7. Conclusions

In addition to DG, PA is a versatile lipid second messenger. It was recently demonstrated that DGK isozymes selectively generate various PA species, which are independent of PI turnover, in isozyme-dependent and cell/stimulation-dependent manners. Moreover, there are a number of PABPs and several of them exhibit PA species selectivity. In addition, the lists of DGK isozyme-derived PA species and PABPs, especially PA species-selective PABPs, are still growing. Because PA species selectivity of only a small part of identified mammalian PABPs has been determined, the selectivity of other PABPs should be re-evaluated to explore the functions of PA species/PABPs in more detail. Most likely, many of them would show their own PA selectivity. Therefore, the recent progress in DGK and PABPs allows us to speculate that the DGK–PA–PABP axes may configure a massive network that is more complex and larger than we expect.

However, there are still many questions concerning PA and PABPs. For example, why does a variety of PA species and PABPs exist? Do PABPs most efficiently recognize fatty acid compositions? What are the upstream DG supply pathways for DGK isozymes lacking the SAM domain? Hence, we may still be in the dark in terms of PA molecular species, their generating pathways, and their molecular functions. However, there is no doubt that diversities of PA species and PABPs are key to exploring molecular mechanisms of a variety of physiological and pathological events regulated by DGK isozymes (and PLD/LPAAT).

## Figures and Tables

**Table 1:** PABPs and their PA species selectivity.

Organism	Protein	PA Species									Affinity (*K*d)/ PA Species	PA-Binding Region
		16:0/16:0	16:0/18:1	18:1/18:1	18:0/18:1	18:0/18:0	18:0/20:4	18:0/22:6	Other	Mixture		
**Mammal**	**Raf-1** **(C-Raf)**	**50**	–	75	–	–	–	–	100 (16:1/18:1)	–	78 nM/ 18:1/18:1-PA	FRNEVAVLRKTRHVNILLFMGYMTKDNLAIVTQWCEG
	PKC	–	–	–	–	–	–	–	–	100	–	KLKLIPDPKNESKQKTKTIRSTLN
	PKC	–	–	–	–	–	–	–	–	100	–	–
	PKC	–	100	–	–	–	–	–	–	–	20–313 µM/ 16:0/18:1-PA	SLKPTAWSLRHAVGPRPQTF……VFHDAPIGYDDFVA (part of C2 domain)
	PKC	–	–	–	–	–	–	–	–	100	–	–
	PKN	–	–	–	–	–	–	–	–	100	15.9 µM/ PA mixture	–
	mTOR	–	–	–	–	–	–	–	–	100	–	RNVKGMFEVLEPLHAMMERGPQTLKETSFNQAYGRDLMEAQEWCRKYMKSGNVKDLTQAWDLYYHVFR
	mTORC2	100	0	0	–	–	–	–	–	–	–	–
	Akt	–	–	–	–	–	–	–	100 (16:0/20:4)	–	–	PH domain
	PAK1	–	–	–	–	–	100	–	–	100	–	–
	p70S6K1	–	100	–	–	–	–	–	–	–	–	–
	Fer	–	–	–	–	–	–	–	–	100	–	SMERKERLSK FESIRHSIAG
	GRK	–	–	–	–	–	–	–	–	100	–	–
	LATS1	100	–	–	–	–	–	–	–	–	–	MOB binding domain, aa 601–751
	KSR1	75	-	75	-	-	-	-	100 (16:1/18:1)	-	1.2 µM/ 18:1/18:1-PA	FKKEVMNYRQTRHENVVLFMGACMNPPHLA
	PIP5K	–	–	–	–	–	–	–	–	100	–	–
	PIP5KI	–	100	–	–	–	–	–	–	–	–	KPERDVLMQDFYVVESIFFPSEGSNLTPAHHFQ
	SphK1	100	–	–	–	–	–	–	–	–	–	–
	DGK	–	–	100	–	30	100	–	40 (14:0/14:0)	100	6–13 pmol/ 18:1/18:1-PA (Overlay)	RVH domain and EF-hand motifs, aa 1–259
	CKM	86	57	93	–	100	14	14	–	–	2.0 µM/ 16:0/16:0-PA	–
	SHP-1	100	–	–	–	–	–	–	–	–	0.04 μM/ 16:0/16:0-PA	SSKHKEDVYENLHTKNKREEKVKKQRSADKEKSKGSLKRK
	PP1c	–	–	100	–	–	–	–	–	–	1.37 µM/ 18:1/18:1-PA	GAMMSVDETLMCSFQILKPAEKKKPNATRPVTPPRGMITKQAKK
	Synaptojanin-1	20	30	40	–	30	100	100	–	–	–	–
	Lipin1	–	–	–	–	–	–	–	–	100	–	VVKKRRKRRRKSQLDSLKR
	PLC 1	89	–	–	–	88	–	–	100 (14:0/14:0)	100	15 mol%/ PA mixture	C-terminus, aa 944-955
	PLC 1	–	–	–	–	–	–	–	–	100	–	–
	PLC 3	–	–	–	–	–	–	–	–	100	–	–
	PLC	–	–	–	–	–	–	–	–	100	160 µM/ PA mixture	–
	Sos	–	–	–	–	–	–	–	–	100	0.2–0.5 µM/ PA mixture	HF domain, aa 97–99 (RKR); PH domain, aa KSNHGQPRLPGA
	RA-GEF-1/2 (PDZ-GEF)	–	–	–	–	–	–	–	–	100	–	CDC25 homology domain, aa 919–967; PDZ domain, aa K428 and R429
	Epac1	–	–	–	–	–	–	–	–	100	–	RDRKYHLRLYRQCCSGR
	DOCK1	–	–	–	–	–	–	–	–	100	–	C-terminal domain, aa 1610–1698
	DOCK2	–	–	–	–	–	–	–	–	100	–	EYGVREMPDFEDRRVGRPRSMRSKKRT
	RasGAP	–	–	–	–	–	75	–	100 (18:2/18:2)	–	–	–
	NF1	–	–	–	–	–	100	–	–	–	12 µM/ 18:0/20:4-PA	–
	RacGDI	–	–	100	–	–	100	–	–	–	–	–
	1-Chimaerin	–	–	–	–	–	–	–	–	100	–	–
	2-Chimaerin	–	–	–	–	–	–	–	–	100	–	–
	AGAP1	–	–	–	–	–	–	–	–	100	–	GTP binding protein-like domain, adjacent to PH domain
	ASAP1	–	–	–	–	–	–	–	–	100	–	–
	ACAP1/2	–	–	–	–	–	–	–	–	100	–	–
	ArfGAP1/2	–	–	–	–	–	–	–	–	100	–	–
	RA-RhoGAP	–	–	–	–	–	–	–	–	100	–	RAELKRGLQRQERHLFLFND
	RGS4	–	–	–	–	–	–	–	–	100	8 mol%/ PA mixture	N-terminal 57 aa
	Sin1	–	–	–	–	–	–	–	–	100	–	PH domain
	Arf1/6	–	–	–	–	–	–	–	–	100	–	–
	Rac1	–	–	–	–	–	–	–	100 (14:0/14:0)	–	–	AVLCPPPVKKRKRKCLLL
	Drp1	85	–	100	–	–	–	–	–	–	–	Stalk domain TAKYIETSEL
	PDE4A1	–	100	77	85	–	–	38	69 (16:0/18:2)	–	6.8 µM/ 18:0/18:1-PA	PWLVGWWDQFKR (requires calcium)
	PDE4A5	100	23	–	18	53	0	27	–	–	1.44 µM/ 16:0/16:0-PA	–
	PDE4B1	–	–	–	–	–	–	–	–	100	–	–
	PDE4D3	–	–	–	–	–	–	–	–	100	–	DPMTSPGSGLILQANFVHSQRRESFLYRS
	PDE4	–	74	100	78	–	94	–	–	89	–	–
	Praja-1	–	<5	25	–	25	13	100	–	–	–	–
	p47*phox*	–	100	–	–	–	–	–	–	–	1.2 nM/ 16:0/18:1-PA	VYRRFTEIYEFHKTLKEMFPIEAGAINPENRIIPHLPAPKWFD (part of PX domain)
	-Synuclein	19	32	100	–	<5	32	–	–	–	6.6 µM/ 18:1/18:1-PA	N-terminal 60 aa
	LDHA	44	22	22	22	100	100	100	–	–	3.7 µM/ 18:0/22:6-PA	–
	NF2	100	–	–	–	–	–	–	–	–	–	–
	Seipin	<50	100	–	–	<50	–	–	–	100	–	C2-like domain, aa 60–200
	CIDEA	–	–	–	–	–	–	–	100 (12:0/12:0)	–	–	C-terminal domain, RCTSFKAVLRNLLRFMS
	Bazooka (Par-3)	100	–	–	–	–	–	–	–	–	–	PDZ2 domain, aa 458–546; PDZ3 domain, aa 635–682
	Dvl-2	100	100	–	–	–	–	–	–	–	–	GLLKAGLIRHTVNKITFSEQ
	RPK118	–	–	–	–	–	–	–	–	100	–	KRYSDFKKLHKELW
	Neurogranin	100	–	–	–	–	–	–	–	100	–	IQ motif, aa 29-47
	-COP	–	–	–	–	–	–	–	–	100	–	–
	NSF	–	–	–	–	–	–	–	–	100	–	–
	Kinesin	–	–	–	–	–	–	–	–	100	–	–
	Anc	–	–	<5	–	–	–	–	–	100	–	–
	Nir	–	–	–	–	–	–	–	–	100	–	IVAGYGSPKDVAVYAALGLSPSQTYIVGR
	IQGAP1	–	–	100	–	–	–	–	–	–	–	KKEKIQTGKK
	CIN85	–	–	–	–	–	–	–	–	100	–	LDEEKKIRLRLQMEVNDIKK
	Syntaxin1A	100	-	-	-	-	-	-	-	-	EC50 2.0 pmol/ 16:0/16:0-PA (Overlay)	DTKKAVKYQSKARRKKIMI
	CgA	–	–	–	100	–	–	100	–	–	–	–
Insect	LKB1	–	–	–	–	–	–	–	–	100	–	VKKKGSALKRRAKKLTSCISVRKLSHCRTS
	TREK-1	–	–	–	–	–	–	–	–	100	15.7 µM/ PA mixture	–
Yeast	Pah1	~100	~100	~100	100	–	–	–	~100 (16:0/18:2) ~100 (18:0/18:2)	–	*K*m 0.65 mol%/ 18:1/18:1-PA	–
	Opi1p	–	100	83	83	–	–	75	88 (16:0/18:2)	100	4.5 µM/ 18:0/18:1-PA	KRQKLSRAIAKGKDNLKEYKLNMSIESKKR
	Spo20p	–	87	80	100	–	–	72	67 (16:0/18:2)	–	2.2 µM/ 18:0/18:1-PA	RLHVKLKSLRNKIHKQLH
	Sso1p	–	100	–	–	–	–	–	–	–	–	KAVKSARKARRNKIRCWLIV
	Sec18p	–	100	–	–	–	–	–	–	–	1.4 µM/ 8:0/8:0-PA	ATP binding site (D1–D2 domain)
	Ups1	–	100	–	–	–	–	–	–	–	–	R25, K61, K155, I78, V106
	Chm7	–	100	–	–	–	–	–	–	–	–	RKGFAKAARSAKESTNMYKSRK
Plant	ABI1	0	–	100	–	10	–	–	–	–	–	ESRKVLISRINSPNLMKESAAADIVVVDISAG
	PP2CA	10	–	100	–	10	–	–	100 (18:2/18:2)	100	–	–
	TGD2	–	–	–	–	–	–	–	–	100	39.6% (w/w) PA mixture	aa 201–225
	TGD4	–	–	100	–	–	–	–	–	–	–	aa 1–80, 110–145
	AtPDK1	–	–	–	–	–	–	–	–	100	–	PH domain aa 391–491
	MKK7/9	<10	70	70	50	30	–	–	–	100	–	–
	SnRK2.4	–	100	–	–	–	–	–	–	–	–	aa 261–302
	PID	0	30	30	<5	0	–	–	100 (18:2/18:2)	30	–	LALKKKMHR
	AtSphK1	–	–	–	–	–	–	–	–	100	0.3 µM/ PA mixture	VSGDGI
	RGS1	–	–	–	–	–	–	–	–	100	0.3 mM/ PA mixture	PLLSQISLKK
	PEPC	–	–	–	–	–	–	–	–	100	–	–
	LHY	80	30	–	–	–	–	–	–	100	0.18 µM/ 16:0/16:0-PA	–
	CCA1	100	30	–	–	–	–	–	–	30	0.12 µM/ 16:0/16:0-PA	–
	Werewolf	–	100	100	–	–	–	–	100 (18:2/18:2)	100	3.99 µM/ 18:1/18:1-PA	RIAKKTGLKRCGKSCRLRWMNYL
	AHL4	0	100	100	–	0	–	–	–	–	–	–
	AKT2	<10	<10	<10	<10	<10	–	–	100 (16:0/18:2)	–	–	aa 317–855
	MAP65-1	<5	50	50	30	<5	–	–	100 (18:2/18:2)	100	–	ARILVSKIPAM
	RbohD160	<5	100	100	–	<5	–	–	100 (16:0/18:2)	100	–	SRELRRVSFRRPSPAVRRFDR
	14-3-3 protein	–	–	–	–	–	–	–	–	100	EC50 48.5 µM/ PA mixture	LSVAYKNVIGARRASWRIIS
	MtDef4	100	100	100	–	–	–	–	0 (8:0/8:0)	–	–	RGFRRR
	NsD7	–	–	–	–	–	–	–	–	100	–	DGHCSKILRR
	SNX	–	–	100	–	–	–	–	–	–	–	–

PA species that most strongly bind to each PABP are set to 100. PABPs that have PA species selectivity are indicated in red, and the preferred PA species are highlighted in red font. –, not determined. Amino acid residues that are critical for PA recognition are underlined. References of PABPs listed: *Mammal*: Raf-1 (C-Raf) [18,19,20], PKCα [125,126,127], PKCδ [128], PKCε [21,22], PKCζ (aPKC) [23,129], PKN [130], mTOR [24], mTORC2 [131], Akt [132,133], PAK1 [134], p70S6K1 [135], Fer [136], GRK [137], LATS1 [138], KSR [139], PIP5K [25,26], PI(4)P5KIγ [140], SphK1 [27], DGKγ [141], CKM [67], SHP-1 [142], PP1c [28], synaptojanin-1 [Hoshino, F. and Sakane, F., unpublished work], lipin1β [29], PLCβ1 [143,144,145], PLCγ1 [30], PLCδ3 [146], PLCε [147], Sos [148], RA-GEF-1/2/PDZGEF [149,150], Epac1 [151], DOCK1 [152], DOCK2 [153], RasGAP [31], NF1 [154], RacGDI [155], α1-chimaerin [32], β2-chimaerin [156], AGAP1 [157], ASAP1 [158], ACAP1/2 [159], ArfGAP1/2 [160], RA-RhoGAP [161], RGS4 [162,163], Sin1 [164], Arf1/6 [34], Rac1 [35,36], Drp1 [165], PDE4A1 [37,38], PDE4A5 [166], PDE4B1 [39], PDE4D3 [167], PDE4 [168], Praja-1 [66], p47*^phox^* [41], α-synuclein [42,43], LDHA [68], NF2 [138], seipin [169], CIDEA [170], Bazooka/Par-3 [171], Dvl-2 [172], RPK118 [173], neurogranin [174], β-COP [34], NSF [34], kinesin [34], Anc [175], Nir [176], IQGAP1 [177], CIN85 [178], syntaxin1A [179], CgA [180]. *Insect*: LKB1 [181], TREK-1 [182]. *Yeast*: Pah1 [183], Opi1p [38,184], Spo20p [38,185,186], Sso1p [187], Sec18p [188,189], Ups1 [190,191], Chm7 [192]. *Plant*: ABI1 [193], PP2CA [194], TGD2 [195,196], TGD4 [197], AtPDK1 [198], MKK7/9 [199], SnRK2.4 [200], PID [201], AtSphK1 [202], RGS1 [203], PEPC [204], LHY [205], CCA1 [205], Werewolf [206], AHL4 [207], AKT2 [208], MAP65-1 [209], RbohD160 [210], 14-3-3 protein [211], MtDef4 [211], NsD7 [212], SNX [213].

## Abbreviations

Arf	ADP-ribosylation factor
aPKC	Atypical protein kinase C
BPD	Bipolar disorder
CDP-DG	Cytidine diphosphate diacylglycerol
CL	Cardiolipin
CKM	Creatine kinase-muscle type
CPE	Ceramide phosphoethanolamine
cPKC	Conventional protein kinase C
DG	Diacylglycerol
DGBP	Diacylglycerol-binding protein
DGK	Diacylglycerol kinase
DHA	Docosahexaenoic acid
ER	Endoplasmic reticulum
FMR	Fragile X mental retardation
FXS	Fragile X syndrome
GRP	Guanyl nucleotide-releasing protein
GWAS	Genome-wide association study
KO	Knockout
LC	Liquid chromatography
LDH	Lactate dehydrogenase
LPAAT	Lysophosphatidic acid acyltransferase
MARCKS	Myristoylated alanine-rich C-kinase substrate
MS	Mass spectrometry
mTOR	Mammalian target of rapamycin
mTORC	mTOR complex
MUFA	Monounsaturated fatty acid
nPKC	Novel protein kinase C
OCD	Obsessive-compulsive disorder
PA	Phosphatidic acid
PABD	Phosphatidic acid-binding domain
PABP	Phosphatidic acid-binding protein
PAP	PA phosphatase
PC	Phosphatidylcholine
PDZ	Postsynaptic density 95, discs large, zonula occludens-1
PE	Phosphatidylethanolamine
PG	Phosphatidylglycerol
PI	Phosphatidylinositol
PIP_2_	Phosphatidylinositol 4,5-bisphosphate
PIP5K	PI-4-phosphate-5-kinase
PH	Pleckstrin homology
PKC	Protein kinase C
PKD	Protein kinase D
PLC	Phospholipase C
PLD	Phospholipase D
PS	Phosphatidylserine
PUFA	Polyunsaturated fatty acid
RA	Ras-associated
RVH	Recoverin homology
SAM	Sterile α motif
SFA	Saturated fatty acid
SphK	Sphingosine kinase
SMSr	Sphingomyelin synthase-related protein
Spo20p	Sporulation-specific protein 20
T2D	Type 2 diabetes
